# Bioassay-Guided Fractionation of Extracts from *Codiaeum variegatum* against *Entamoeba histolytica* Discovers Compounds That Modify Expression of Ceramide Biosynthesis Related Genes

**DOI:** 10.1371/journal.pntd.0002607

**Published:** 2014-01-09

**Authors:** Emmanuel Mfotie Njoya, Christian Weber, Nora Adriana Hernandez-Cuevas, Chung-Chau Hon, Yves Janin, Melanie F. G. Kamini, Paul F. Moundipa, Nancy Guillén

**Affiliations:** 1 University of Yaoundé I, Faculty of Science, Department of Biochemistry, Laboratory of Pharmacology and Toxicology, Yaoundé, Cameroon; 2 Institut Pasteur, Cell Biology of Parasitism Unit, Paris, France; 3 INSERM U786, Paris, France; 4 Institut Pasteur, Unité de Chimie et Biocatalyse, Paris, France; McGill University, Canada

## Abstract

Leaves of *Codiaeum variegatum* (“garden croton”) are used against bloody diarrhoea by local populations in Cameroon. This study aims to search for the active components from *C. variegatum* against *Entamoeba histolytica*, and thereby initiate the study of their mechanism of action. A bioassay-guided screening of the aqueous extracts from *C. variegatum* leaves and various fractions was carried out against trophozoites of *E. histolytica* axenic culture. We found that the anti-amoebic activity of extracts changed with respect to the collection criteria of leaves. Thereby, optimal conditions were defined for leaves' collection to maximise the anti-amoebic activity of the extracts. A fractionation process was performed, and we identified several sub-fractions (or isolated compounds) with significantly higher anti-amoebic activity compared to the unfractionated aqueous extract. Anti-amoebic activity of the most potent fraction was confirmed with the morphological characteristics of induced death in trophozoites, including cell rounding and lysis. Differential gene expression analysis using high-throughput RNA sequencing implies the potential mechanism of its anti-amoebic activity by targeting ceramide, a bioactive lipid involved in disturbance of biochemical processes within the cell membrane including differentiation, proliferation, cell growth arrest and apoptosis. Regulation of ceramide biosynthesis pathway as a target for anti-amoebic compounds is a novel finding which could be an alternative for drug development against *E. histolytica*.

## Author Summary

Amoebiasis is a disease caused by a protozoan parasite, *Entamoeba histolytica*, with or without clinical symptoms. Humans are the only relevant host of this parasite, which mainly targets the large intestine and the liver. The current drug, metronidazole, has been successfully used against this parasite for several years. However, some reports have shown either parasite resistance or adverse effects due to its long term usage. Our study thereby pointed to alternative treatment of this infection by investigating the rational use of *Codiaeum variegatum* also referred as “garden croton” which is a medicinal plant used in Cameroon against bloody diarrhoea. We moved into the identification of the most efficient fraction of the aqueous extract of this plant, and initiated the characterization of the mechanism of action of this fraction. Upon treatment with the active fraction, parasite death occurs within two days through morphological changes such as cell membrane disorganization and cell destruction. More deeply, we found that components of the active fraction modify expression of genes involved in ceramide biosynthesis, a pathway responsible for cell death and growth inhibition. Our study therefore suggests a novel finding which could be further explored for screening of anti-amoebic drugs.

## Introduction

Medicinal plants are recognized by the World Health Organization as alternatives in the treatment of various diseases and the interest of health professionals for medicinal plants is increasing everyday [1]. Medicinal plants contain a variety of secondary metabolites, which can be used to prevent or cure diseases, or to promote general health and well-being [2],[3]. During the last decades, scientific evidences of the medicinal values of plant products (through *in vitro* investigation) aroused public concerns about the conservation of such plants, in order to retain their economic and therapeutic significance [4]. Modern pharmaceutical industry relies mainly on the diversity of secondary metabolites in medicinal plants for the discovery of new compounds with novel biological properties. It is estimated that natural products and their derivatives and analogues represent over 50% of all drugs in clinical use [5],[6]. Therefore, further evaluation of drugs derived from plants requires the screening of large numbers of plant extracts, isolation and identification of the active compounds, the study of their mechanism of action, as well as the proof of its non-toxicity to human cells. In Cameroon, biodiversity is an important source of bioactive natural compounds and exploration of this biodiversity, based on ethnopharmacology approach from traditional healers, represents a promising strategy to fight against diseases such as intestinal infections. We have focused on amoebiasis, a human infectious disease caused by the amoebic parasite *Entamoeba histolytica*, which mainly targets intestine and liver. Humans are the only relevant host of this parasite and infection occurs upon ingestion of contaminated water or food containing cysts forms of *E. histolytica*. In rural areas of some developing countries, up to 20% of the population is infected with *E. histolytica*. Trophozoites (the vegetative form) are released in the intestine by excystation. Once the trophozoite has penetrated the intestinal mucosal layer, it induces intestinal amoebiasis responsible of colitis and bloody diarrhoea [7],[8]. An acute immune response is triggered during the early stages of the parasite infection. Amoebic liver abscesses are the most frequent with severe extra-intestinal clinical manifestations of amoebiasis [8]. The morbidity and the economic impact of dysentery make of amoebiasis a prominent public health problem.

Metronidazole (MTZ) is the drug of choice used in clinical practice for the treatment of amoebiasis since 45 years [9]. This drug has been reported to be potentially carcinogenic to humans due to the facts that it is mutagenic in bacterial systems, genotoxic to human cells and carcinogenic to animals [10]. Also, a significant increase in DNA damage was found in lymphocytes from healthy subjects one day after treatment with therapeutic doses of MTZ [11]. Moreover, the current use and long term treatment with MTZ is responsible for many side effects such as toxic symptoms of metallic taste, headache and dry mouth and to a lesser extent nausea, glossitis, urticaria, pruritus, urethral burning and dark colored urine [12]. Some clinical isolates of *E. histolytica* present diverse susceptibility to MTZ [13] and drug resistance has been induced in axenic cultures of amoebic virulent strains following continuous exposure to increasing MTZ concentrations [14]. MTZ resistance in *E. histolytica* has been associated to increased expression of iron-containing superoxide dismutase and peroxiredoxin and decreased expression of flavin reductase and ferredoxin 1, which are enzymes involved in redox pathways of *E. histolytica* [14]. Recent data report thiol-containing redox proteins (Thioredoxin and Thioredoxin Reductase) covalently modified and inactivated by MTZ [15],[16]. To provide alternatives to MTZ clinical uses, several efforts are underway. Recently, a screening of US Food and Drug Administration (FDA)-approved drugs identified auranofin as active against *E. histolytica* in culture and also in a mouse model of amoebic colitis or in a hamster model of amoebic liver abscess [17]. Auranofin, is a US FDA approved drug used for treatment of rheumatoid arthritis. This drug is reported to be an inhibitor of thioredoxin reductase (TrxR) thereby suggesting that *E. histolytica* TrxR is likely its target [17]. Additionally, a common reaction to auranofin is persistent diarrhoea reported in 50% of patients, a fact precluding its use for amoebiasis treatment. A galacto-glycerolipid isolated from *Oxalis corniculata* has shown a strong anti-amoebic activity with no effect on intestinal microbial flora or on the mammalian cell line HEK-293 [18], despite the fact that the mechanism of its action is still unclear. Fifty-five medicinal plants belonging to different families, selected on the basis of their traditional use against intestinal and liver disorders, were tested for their anti-amoebic activities on polyxenic cultures of *E. histolytica*. From these plants, the aqueous extract of leaves of *C. variegatum* exhibited a pronounced anti-amoebic activity [19]. Identification of its active compounds and characterization of their mechanism of action might provide new candidates for development of anti-amoebic drugs. The present study goes further in the characterization of *C. variegatum* amoebicide fraction. A bioassay-guided screening of various fractions of the aqueous extracts from *C. variegatum* leaves was carried out against trophozoites in axenic culture. The EC_50_ values were determined for toxic fractions inducing parasite death. Then, an attempt to understand the mode of action of the compounds within active fraction was addressed by differential gene expression analysis using high-throughput RNA Sequencing. The data suggested a multi-target mechanism of action inducing cell death through the disturbance of lipid metabolism in the parasite. This cellular response is different when compared to results obtained by MTZ treatment. We therefore hypothesize that cell death might occur through the disturbance of certain biochemical processes within the cell membrane involving ceramide, a bioactive lipid known as cellular signal implicated in induction of apoptosis and cell growth inhibition. This study suggests a new mechanism of action for anti-amoebic compounds, which can be further explored as a strategy for drug development.

## Methods

### Preparation of extracts

Leaves of *C. variegatum* were harvested in Yaoundé (Cameroon) according to several criteria: sites of cultivation (forest and garden), period of the day (morning, midday, afternoon and midnight) and stage of development (young and old leaves). It should be noted that young or old leaves as well as leaves from the forest and the garden were all collected in the morning. Leaves from more than 20 different plants were collected. The leaves were thoroughly washed with tap water, rinsed with distilled water, dried at room temperature and ground. The powder obtained, 200 grams (g) for each batch, was mixed with 2 litres of distilled water for the preparation of aqueous extracts by decoction for 1 hour. After filtration with Whatman No. 1 filter paper, the filtrate collected was dried by lyophilization.

### Fractionation procedure and isolation of compounds

**Figure 1:**
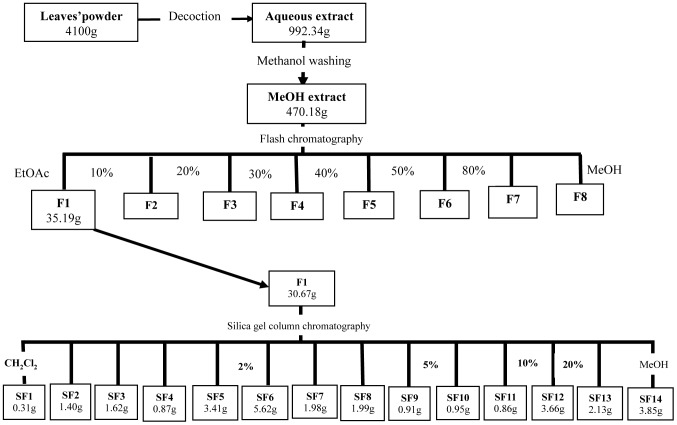
Fractionation process of the aqueous extract of *Codiaeum variegatum*. Activity-guided purification of fractions and sub-fractions using flash chromatography followed by silica gel column chromatography of the ethyl-acetate fraction (F1), the highest anti-amoebic fraction. Note that active fractions and sub-fractions are marked in bold in the following scheme.

The sequential decoction of powdered leaves (start with 4100 g) yielded 992.34 g of aqueous extract which after washing with methanol led to 470.18 g extract. The methanol extract was then partitioned on silica gel by flash chromatography using a gradient of ethyl acetate (EtOAc) and methanol (MeOH). Eight stages of polarity were used: EtOAc (Fraction 1); EtOAc/MeOH 10% (Fraction 2); EtOAc/MeOH 20% (Fraction 3); EtOAc/MeOH 30% (Fraction 4); EtOAc/MeOH 40% (Fraction 5); EtOAc/MeOH 50% (Fraction 6); EtOAc/MeOH 80% (Fraction 7) and MeOH (Fraction 8). Fraction 1 (30.67 g) was further partitioned with a gradient polarity of methylene chloride (CH_2_Cl_2_)/methanol (MeOH) solvent using a silica gel column chromatography. Four solvent systems (CH_2_Cl_2_; CH_2_Cl_2_/MeOH 2%; CH_2_Cl_2_/MeOH 5% and CH_2_Cl_2_/MeOH 10%) were used during the elution and more than 100 samples (80 ml each) were collected and grouped in 14 sub-fractions according to their chemical profiles analysed by thin layer chromatography. In all the fractionation, solvent was removed in collected samples by using a rotary evaporator. The final fraction or powder was stored at 4°C. Figure 1 depicts the general scheme of fractionation.

### *In vitro* culture of *E. histolytica*

The virulent strain of *E. histolytica* (HM1:IMSS) was grown in 15 ml screw cap glass tubes at 37°C on TYI-S-33 axenic medium supplemented with 15% (v/v) complement-inactivated bovine serum (PAA laboratories GmbH, Austria), 3% Diamond vitamin Tween 80 (Sigma-Aldrich, Saint Quentin Fallavier, France) and 1% Penicillin-Streptomycin (Sigma-Aldrich, Saint Quentin Fallavier, France) [20]. The culture medium was renewed twice in a week and trophozoites at the exponential phase of growth were used in all experiments.

### *In vitro* anti-amoebic assay

Plant extracts, fractions, sub-fractions and isolated compounds were prepared using sterile DMSO (Sigma-Aldrich, Saint Quentin Fallavier, France) and culture medium leading to concentrations of 100, 50 or 10 mg/ml respectively. Each mixture was filtered with sterile syringe filters (Ø 22 µm) and aliquots of two-fold serial dilutions were prepared from these stock solutions. A fresh culture of 5×10^3^ trophozoites per milliliter was introduced in each well of the 48 well microtiter plate and after allowing the parasite to adhere at the bottom of the well, 5 µl of the tested extract or compound was added. The concentration of DMSO did not exceed 0.5% in all assays performed. Each test included a blank (medium only) and two controls (one consist of trophozoites with medium only and the other consist of trophozoites with medium containing DMSO). Metronidazole (Sigma-Ultra, CA, USA) was used as the positive control in each assay (see tables and figures in the results section for the concentrations tested). For microarray experiments, MTZ was used at 8 µg/ml, which correspond to 50 µM. The plates were introduced in Genbag anaer (Biomerieux, Marcy l'Etoile, France) and incubated for 48 to 72 hours at 37°C and the cell viability was evaluated with a hemocytometer using the trypan blue (Sigma-Aldrich, Saint Quentin Fallavier, France) exclusion technique. In some cases (as indicated in the tables) cells were incubated only for 24 hours. The mortality rate of trophozoites was calculated for each concentration tested according to the formula below. The 50% efficient concentrations (EC_50_) were determined by plotting the graph of mortality rate versus the concentration tested and using a normalised sigmoidal function of the software *Statgraphics, Plus Version 5.0*.
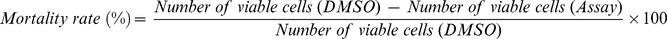



### Cytotoxicity assay on human enterocytes from the Caco-2 cell line

The human colon carcinoma cell line TC7 (Caco-2) was grown to 21 days confluence in Dulbecco's modified Eagle's medium (Life Technologies, Saint Aubin, France) supplemented with 15% fetal calf serum (Eurobio, Les Ulis, France) and 1% non-essentials amino acids (Life Technologies, Saint Aubin, France) at 37°C in a 10% CO_2_ incubator. Differentiated Caco-2 cells were incubated for 48 hours with varying concentrations of ethyl acetate fraction (F1), sub-fractions (SF9, SF10, SF11 and SF9B) and the aqueous extract. The highest concentration tested on these cells was 1 mg/ml; over this concentration the aqueous extract was no longer dissolved in DMSO. Staurosporine 0.1 µM (a chemical which induces apoptosis) was used as a control for cell death and the solvent (DMSO) was the control for viability. After incubation, the culture medium was removed and the viability count was performed using the trypan blue exclusion technique.

### Statistical analysis

The tests were performed in triplicate and all data are presented as mean ± SD (standard deviation) values. Statistical analysis was performed using *GraphPad Instat* and student's *t*-test was used to determine *P*-values for the differences observed between test compounds and control. Results were considered significantly different when *P*≤0.05.

### Generation of transcriptome sequencing data

Trophozoites of *E. histolytica* (approximately 1×10^6^) grown in 15 ml glass tubes were treated with plant extract sub-fraction SF9B (at EC_50_, discussed later) or DMSO (control) for 12 and 24 hours (in 3 biological replicates, n = 4×3). Total RNA was extracted from these trophozoites using Trizol reagents (Invitrogen, Saint Aubin, France) and poly(A)+ RNA was purified from 10 µg of total RNA using oligo(dT) coated Sera-Mag Magnetic Particles according to manufacturer's instructions (Thermo Scientific, Fremont, USA). PolyA enriched RNA is chemically fragmented to ∼100 bp (Ambion, USA) and purified with RNeasy MinElute Cleanup Kit (Qiagen,Venlo, Netherlands) according to manufacturer's instructions. Strand-specific cDNA libraries (n = 12) were prepared using an RNA ligation protocol based on Illumina TruSeq Small RNA Sample Preparation Kit [21]. Sequencing of these libraries was performed on a HiSeq 2000 (Illumina) in a multiplexed single-ended setting for 50 cycles using TruSeq SR Cluster kit v3 cBot HS and TruSeq SBS kit v3 HS (Illumina). After sequence files generation using CASAVA 1.7 (Illumina), 3′ adapter sequence was trimmed using Cutadapt [22]. These processed short reads data have been deposited in the European Nucleotide Archive (http://www.ebi.ac.uk/ena/data/view/ PRJEB3953).

### Analysis of transcriptome sequencing data

Sequence reads were mapped to *E. histolytica* genome assembly (AmoebaDB v1.7, http://amoebadb.org/amoeba/) using Tophat version 2.0.6 [23] with default parameters. Coding genes differentially expressed in cells treated with SF9B versus control (in triplicates) at 12 or 24 hours were identified using Cuffdiff version 2.0.2 [23] and DESeq version 1.12.0 [24] with default parameters. Coding gene models were based on the bona fide gene models defined in previous work [25]. Differentially expressed genes were defined as genes at ≤5% false discovery rate with ≥2-fold change identified in either Cuffdiff or DESeq analyzes.

### Microarray analysis of *E. histolytica* upon treatment with metronidazole

Trophozoites of *E. histolytica* (4×10^6^) grown in axenic culture and treated or not with metronidazole (at 50 µM for one hour), were lysed with Trizol reagent and total RNA isolated according to the manufacturer's protocol. RNA was analyzed for integrity and the concentration determined by capillary electrophoresis using the Agilent Bioanalyzer 2100 RNA nanochip Assay (Agilent Technologies). Agilent microarrays EH-IP2008, scanning the entire amoebic genome, were used as previously described [26]. Dye swap hybridizations were performed for the three biological replicates leading to a total of 6 competitive hybridizations. The whole data set was submitted to the ArrayExpress database (Accession number: E-MTAB-1763). After pooling data from technical and biological replicates, differential analysis was carried out as published [26] and includes paired Student's *t*-test. The raw *P*-values were adjusted by the Benjamini and Yekutieli method which controls the false discovery rate (FDR) [27]. We considered as being differentially expressed the genes with a Benjamini and Yekutieli *P*-value <0.05 and expression fold change ≥2.

### Indirect immunofluorescence of treated trophozoites of *E. histolytica*

Trophozoites of *E. histolytica* (5×10^3^ ml^−1^) were mixed with 5 µl of the active sub-fractions to obtain final concentrations of EC_50_. The microtiter plate was introduced in Genbag anaer and then incubated at 37°C for 12, 24 and 48 hours and cell viability was evaluated as above described. The same assay was carried out on 8-wells Chamber Slide System (Brumath, France), the chamber was introduced in a Genbag anaer for 12 hours and the morphology of amoebae was examined using indirect immunofluorescence assay. Briefly, amoebae cells were fixed with 500 µl of formaldehyde 3.7% (Thermo scientific, Waltham-MA, USA) for 30 minutes, washed with 500 µl of 3% BSA in PBS and then incubated for 30 minutes at 37°C. The primary antibody Gal/GalNAc lectin (1∶100 diluted in 1% BSA-PBS) was added and the plates incubated for one hour at 37°C in humidified atmosphere. The plates were washed twice with 1% BSA-PBS. The secondary antibody Alexa Fluor 546 goat anti-rabbit IgG (1∶200 diluted in 1% BSA-PBS) was added and incubated for 45 minutes at 37°C. At the end of the experiment, the plates are washed thrice with PBS and the mounting medium Vectashield with nuclear stain DAPI (Vector-ABCYS) was used. For every assay, DMSO is used as negative control. The cells were observed by epifluorescence microscopy (Olympus).

## Results

### *In vitro* anti-amoebic activity of extracts

**Figure 2:**
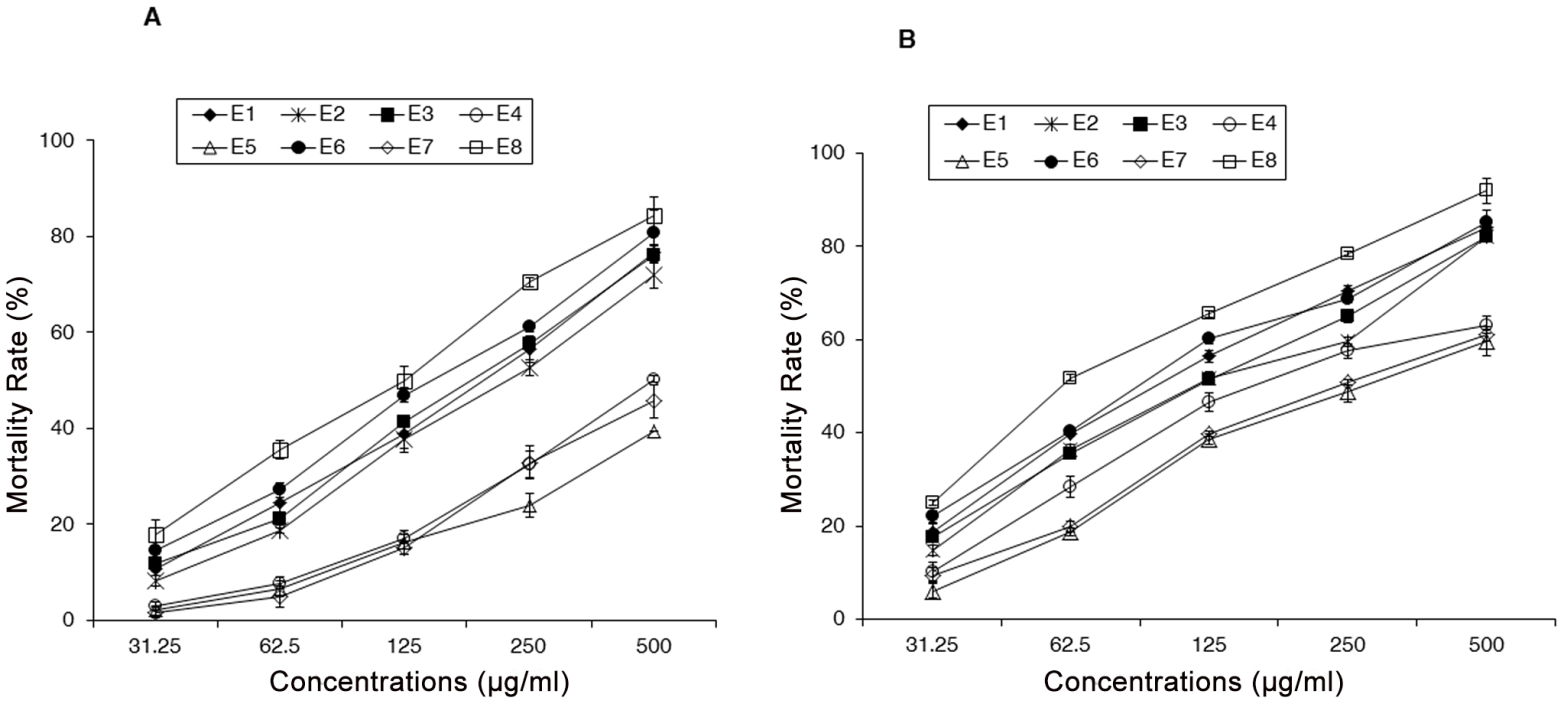
Anti-amoebic activity of different aqueous extracts according collection criteria (E1 = forest; E2 = garden; E3 = morning; E4 = midday; E5 = afternoon; E6 = midnight; E7 = young leaves and E8 = old leaves). Extracts were tested at increasing concentrations (31.25; 62.5; 125; 250; 500 µg/ml) for 48 hours (A) and 72 hours (B) of incubation. Data are obtained from three experiments and are presented as mean with bars as standard deviation (SD) compared to the control (DMSO).

The anti-amoebic action of *C. variegatum* was assessed on axenic culture of trophozoites and the results are presented as follow: in the presence of the plant aqueous extract, the growth inhibition or mortality of *E. histolytica* increases in a concentration dependent manner and the collection criteria of leaves for extract preparation as well as the period of incubation significantly influenced the mortality rate. By plotting the mortality rate against concentration, EC_50_ of the extracts were determined (Table 1). Despite the fact that no extract induced total mortality (100%) in the performed assay, we did not observe any stationary phase in amoebic growth, (Figure 2). We noticed that the anti-amoebic activity of the extracts depends on the leaves harvest criteria and it increases with the time of incubation. No significant difference was observed among the extracts from different sites of plant collection; extract from plants harvested at midnight (E6) showed significantly higher anti-amoebic activity compared to extracts from plants harvested at other period of the day. Extract obtained from old leaves and collected in the morning (E8) exhibited the highest significant anti-amoebic activity amongst all samples tested and displayed a EC_50_ of 120.00 µg/ml after 48 hours of incubation and 60.54 µg/ml after 72 hours of incubation. However, when compared to pure MTZ (EC_50_ = 0.73 µg/ml after 72 hours of incubation), extract E8 showed significantly lower anti-amoebic activity. Due to its complex chemical composition, which may prevent activity of some compounds, we promptly initiated the identification and characterization of active compounds in E8.

**Table 1:** EC_50_ of different aqueous extracts against *E. histolytica* after 48 and 72 hours of incubation.

EC_50_ (Mean ± SD) (µg/ml)		
Incubation time	48 hours	72 hours
E1 (Forest)	204.33±12.02	102.00±4.33
E2 (Garden)	219.00±3.53	118.50±4.35
E3 (Morning)	191.16±5.00	119.33±3.61
E4 (Midday)	491.00±3.53	169.37±0.17
E5 (Afternoon)	N	296.50±7.77
E6 (Midnight)	156.75±3.88	93.16±1.52
E7 (Young)	N	182.50±2.82
E8 (Old)	120.00±7.77^a^	60.54±0.07^a^
Metronidazole	1.38±0.02^a^ ^b^	0.73±0.03^a^ ^b^

^a^ significant difference between extracts;

^b^ significant difference of extracts compared to metronidazole (n = 3, p≤0.05). Extracts E3, E4, E5, E6, E7, E8 were obtained from the forest. N means test non-performed.

### Fractionation of aqueous extract enhances the anti-amoebic activity

After extraction with methanol (MeOH), we attempted to identify the active components in different fractions derived from chromatography (Figure 1). Three fractions (F1, F2 and F8) showed significant anti-amoebic activity, while F1 incubation for 48 hours was the most active amongst all conditions tested. Then, these three active fractions were further tested during 72 hours of incubation. The first observation was that the unfractionated MeOH extract achieved total mortality (100%) at the concentration of 500 µg/ml. Determination of EC_50_ was carried out for the unfractionated MeOH extract and three active fractions, which all exhibited at least 50% mortality (Table 2). The unfractionated MeOH extract was more potent than any of the three active fractions alone, with EC_50_ of 126.50 µg/ml and 53.00 µg/ml after 48 and 72 hours of incubation, respectively. Although the fraction F1 did not achieve total mortality, more than 90% of trophozoites were killed and this fraction was significantly more potent than other two active fractions, with EC_50_ of 202.00 µg/ml and 61.83 µg/ml after 48 and 72 hours of incubation, respectively. F1 was further explored owing to its relatively simpler chemical composition and its relatively higher efficacy compared to the other two active fractions.

**Table 2:** EC_50_ of methanol extract and active fractions against *E. histolytica* after 48 and 72 hours of incubation.

EC_50_ (Mean ± SD) (µg/ml)		
Incubation time	48 hours	72 hours
MeOH Extract	126.50±0.70^a^	53.00±0.50^a^
F1	202.00±3,50^b^	61.83±0.57^b^
F2	398.00±1.83	202.25±0.35
F8	479.50±2.50	234.25±1.06
Metronidazole	1.38±0.02^a^ ^b^ ^c^	0.73±0.03^a^ ^b^ ^c^

^a^ significant difference between methanol extract and fractions;

^b^ significant difference between fractions;

^c^ significance difference of methanol extract and fractions compared with metronidazole (n = 3, p≤0.05).

### Guided anti-amoebic paths to purify the ethyl acetate fraction

**Figure 3:**
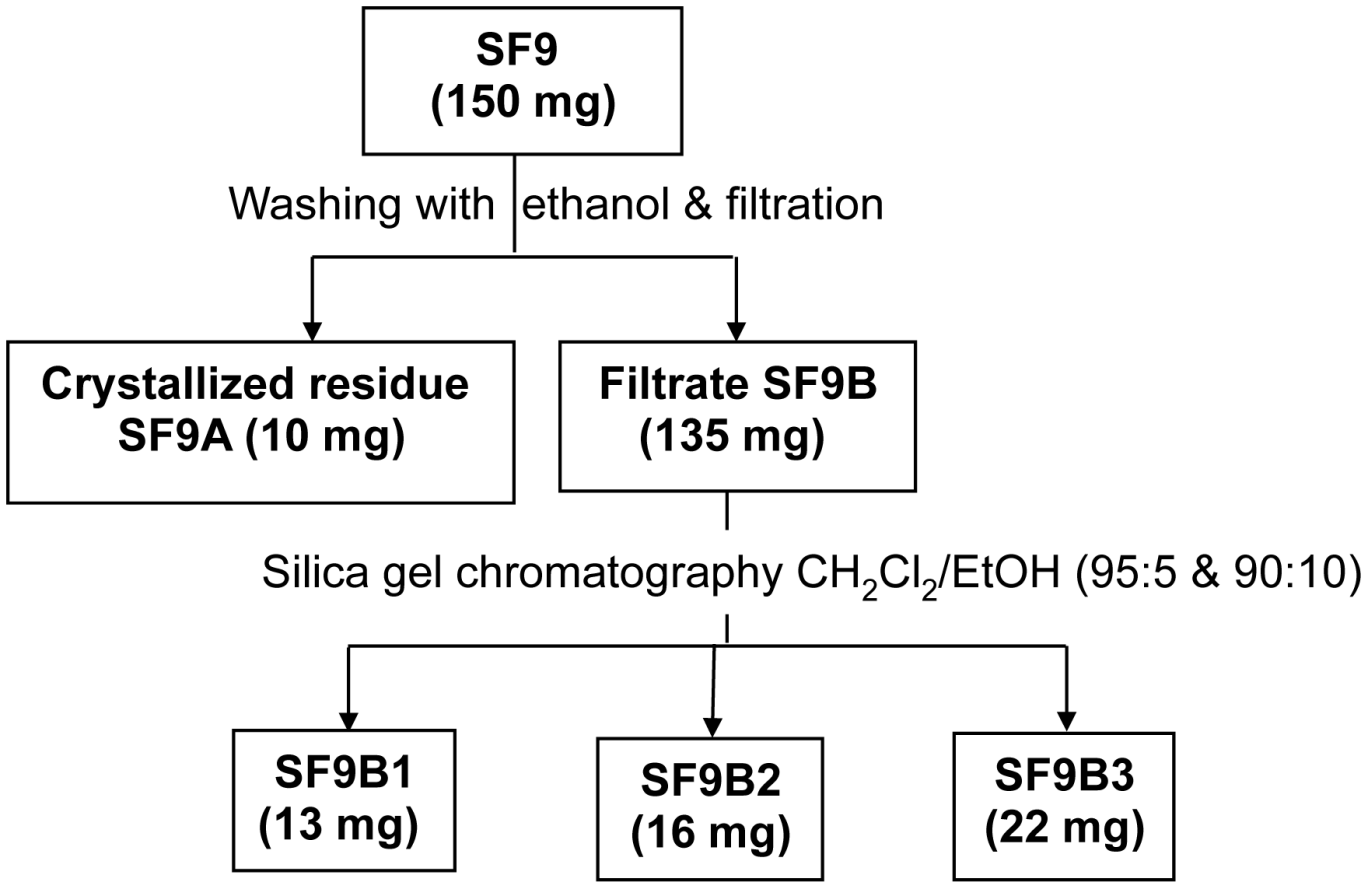
Fractionation process of active sub-fraction (SF9). Activity-guided isolation of compounds using silica gel column chromatography. Active sub-fractions and compounds are marked in bold.

**Figure 4:**
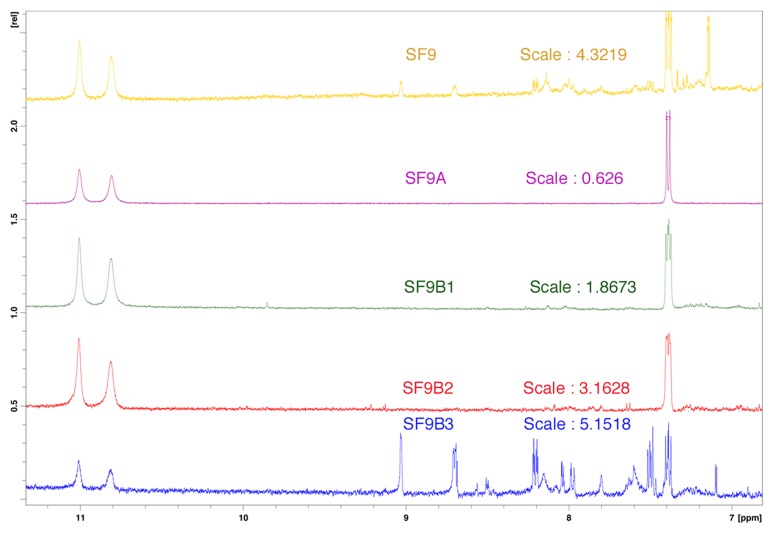
Superposition of nuclear magnetic resonance (NMR) spectra of sub-fractions separated from SF9. (from top to bottom: SF9; SF9A; SF9B1; SF9B2 and SF9B3 respectively). Each product (1–10 mg) was dissolved in 0.7 ml of deuterated DMSO and the mixture was introduced into a tube adapted to the NMR engine and then was subjected to magnetic field to measure these resonances.

**Figure 5:**
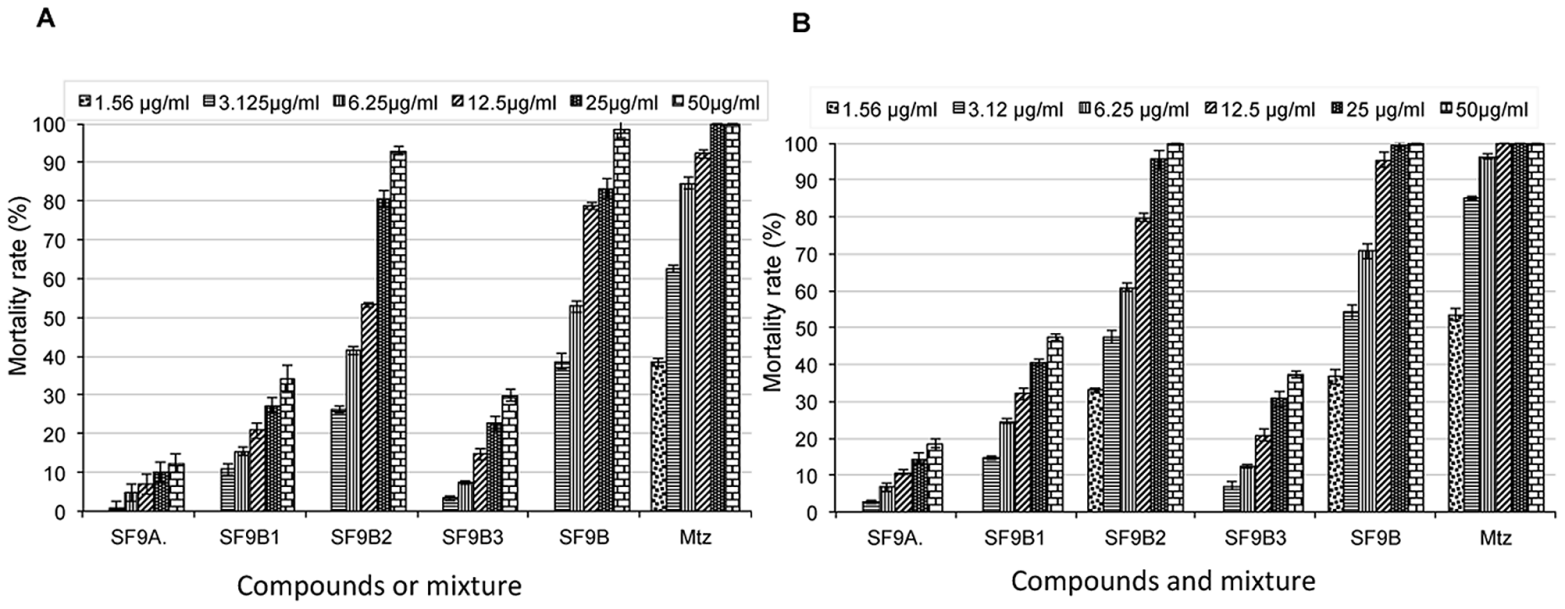
Mortality rate of *E. histolytica* treated with isolated compounds or mixtures compared to metronidazole. Each substance was tested at different concentrations (1.56; 3.125; 6.25; 12.5; 25; 50 µg/ml) respectively after 24 hours of incubation (A) and 48 hours of incubation (B). Data are obtained from three experiments and are presented as mean with bars as standard deviation (SD) compared to the control (DMSO).

Silica-gel column chromatography was performed on F1, yielding 14 sub-fractions grouped according to their frontal ratio on thin layer chromatography profiles (Figure 1). Three of these sub-fractions (SF9, SF10 and SF11) exhibited significant anti-amoebic activity. Table 3 summarises EC_50_ of the active sub-fractions. Mortality due to these sub-fractions increased in a concentration dependent manner and 100% mortality was observed for sub-fractions SF9 at 125 µg/ml and for SF11 at 250 µg/ml after 72 hours of incubation. SF9 showed significantly higher anti-amoebic activity compared to other sub-fractions, with EC_50_≤15.62 µg/ml after 72 hours of incubation. The chemical analysis of the 3 sub-fractions (SF9, SF10 and SF11) using thin layer chromatography and nuclear magnetic resonance revealed a common spot at the same frontal ratio and similarity between the three spectra meaning that these sub-fractions contain similar compounds, respectively. Interestingly, the presence of a small amount of observable crystals in these sub-fractions might imply the existence of certain compounds in the sub-fractions at a considerably high purity. In order to identify these compounds, the most active sub-fraction SF9 was further analysed chemically and the fractionation process is described in Figure 3. In fact, the sub-fraction SF9 was separated into a crystal fraction (SF9A) and a soluble fraction (SF9B). The crystal fraction was unfortunately less efficient than the soluble fraction. Chemical analysis of the soluble fraction suggested that SF9B is likely consisted of mainly 3 compounds (SF9B1, SF9B2 and SF9B3). The comparison of nuclear magnetic resonance (NMR) spectra of these sub-fractions suggests that they contain similar compounds with a common skeleton and some additional chemical groups which are important for their potency. Figure 4 described the superposition of different spectra of isolated sub-fractions or compounds. Further assays showed that SF9B1, SF9B2 and SF9B3 kill trophozoites with different potencies, while SF9B2 exhibited a pronounced activity against trophozoites comparable to the unfractionated soluble fraction (i.e. SF9B) and MTZ. EC_50_ of these active compounds were showed in Table 4. Based on the calculated EC_50_, the soluble fraction SF9B, which contained the mixture of at least 3 compounds, appeared as the most efficient killer of trophozoites compared to any of the isolated compounds alone (Figure 5). The high potency of SF9B can result from synergistic or additional action between isolated compounds and therefore SF9B was used in the following experiments.

**Table 3:** EC_50_ active sub-fractions against *E. histolytica* after 48 and 72 hours of incubation.

EC_50_ (Mean ± SD) (µg/ml)		
Incubation time	48 hours	72 hours
SF9	18.87±1.23^a^	≤15.62^a^
SF10	35.87±4.41	22.87±1.59
SF11	26.50±1.14	17.91±1.46
Metronidazole	1.38±0.02^a^ ^b^	0.73±0.03^a^ ^b^

^a^ significant difference between sub-fractions.

^b^ significance difference of sub-fractions compared to metronidazole (n = 3, p≤0.05).

**Table 4:** EC_50_ of compounds and mixtures against *E. histolytica* after 24 and 48 hours of incubation.

EC_50_ (Mean ± SD) (µg/ml)		
Incubation time	24 hours	48 hours
SF9A	>50.00	>50.00
SF9B	5.71±0.24^a^	2.75±0.10^a^
SF9B1	>50.00	>50.00
SF9B2	10.80±0.35	3.78±0.31
SF9B3	>50.00	>50.00
Metronidazole	2.26±0.03^a^ ^b^	1.38±0.02^a^ ^b^

^a^ significant difference between compounds and mixtures;

^b^ significance difference of compounds or mixtures compared to metronidazole (n = 3, p≤0.05).

### Active components kill trophozoites of *E. histolytica* in axenic culture by inducing morphological changes and cell damage

**Figure 6:**
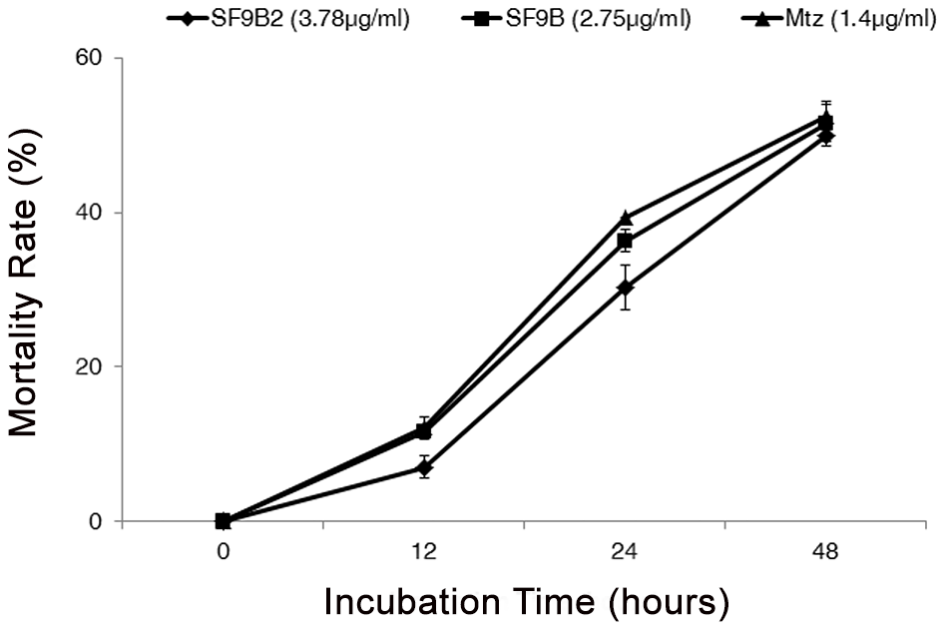
Mortality rate of *E. histolytica* caused by SF9B2, SF9B and metronidazole over time. SF9B2 was tested at 3.78 µg/ml, SF9B at 2.75 µg/ml and MTZ at 1.40 µg/ml for 12, 24 and 48 hours. Data are obtained from three experiments and are presented as mean with bars as standard deviation (SD) compared to the control (DMSO).

After incubating the trophozoites with SF9B for various durations (12, 24 and 48 hours) and concentrations (1.56–50 µg/ml), we determined an optimal condition which is sufficient to visualize cell morphology changes while keeping cell viability. The optimal condition is to expose trophozoites at EC_50_ with SF9B fraction and for a maximum period of 24 hours. The treatment of trophozoites with 3.78 µg/ml of SF9B2 and 2.75 µg/ml of SF9B caused different mortality rate according to the period of incubation (Figure 6). Death of trophozoites is firstly reflected by cell rounding and finally cell lysis. Dying cells detached from the bottom of the microtiter plate and attached viable cells could be counted.

### Viability count of Caco-2 cells incubated with active components

Treatment of Caco-2 cells with the aqueous extract, fraction F1 and sub-fractions SF9, SF10, SF11 and SF9B at a wide range of concentrations did not show significant difference in cell death when compared to the negative control (DMSO). Therefore, at the tested concentrations, extracts and active fractions from *C. variegatum* had no observable cytotoxic effect on Caco-2 cells up to 1 mg/ml, while the positive control (Staurosporine 0.1 µM) induced substantial cytotoxicity between 30–40% of these cells.

### Gene expression changes in trophozoites treated by SF9B

To gain insight into the molecular basis of the anti-amoebic activity of SF9B, a transcriptome analysis was performed using high-throughput RNA sequencing. Briefly, the trophozoites were treated with SF9B at EC_50_ (or DMSO as a control) for 12 and 24 hours in biological triplicates (n = 4×3, see methods). Totally 12 libraries were sequenced and on average ∼13 millions reads (ranging from ∼5 to ∼23 millions) were mapped to the coding genes (n = 7312) in each of the libraries. Differentially expressed genes (DEG) were identified by comparing SF9B treated cells versus control cells of the same time points (i.e. DEG at 12 and 24 hours, see methods for DEG definition). We identified 9 and 30 DEG at 12 and 24 hours respectively (Table 5, Tables S1 and S2). All 9 DEG at 12 hours overlap with DEG at 24 hours and their fold-changes are comparable between time points, suggesting that the transcriptomic changes are generally consistent between time points and progressed in a time-dependent manner. Interestingly, 28 of the 30 DEG were down-regulated upon SF9B treatment, including 9 genes involved in the biosynthesis of ceramide. Ceramide, which is composed of sphingosine and a fatty acid, is found within the cell membrane as a bioactive lipid implicated in a variety of physiological functions including apoptosis and cell growth arrest. These down-regulated DEG includes: acid shingomyelinase (EHI_040600) that mediates production of ceramide from shingomyelin (major lipid in the membrane bilayer), and several genes encoding enzymes involved in production of Acyl-CoA (fatty acid with CoA) or in the synthesis of fatty acid (Table 5). Down-regulation of these genes might imply a diminution of ceramide level in SF9B treated trophozoites. Moreover, two DEG encoding distinct orthologues of the longevity-assurance (Lag) proteins were up-regulated (56% amino acid identity and 72% homology). These two proteins (EHI_139080 and EHI_130860) contain a TLC domain (common to **T**RAM, **L**AG1 and **C**LN8 which are members of a novel family of lipid-sensing proteins), and EHI_139080 in addition carries one ER-targeted motif. The TLC family may possess multiple functions such as lipid trafficking, metabolism, or sensing. Proteins containing TLC domains should catalyse the synthesis of ceramide and in particular Lag1 from *Saccharomyces cerevisiae* is essential for acyl-CoA-dependent ceramide synthesis. According to this function, the upregulation of Lag genes may attempt to overcome the deficiency of ceramide in amoebae treated with SF9B. However, other suggested functions for TLC containing proteins are their role in protecting proteins from proteolysis through their binding to vacuolar ATPases [28] and their function as linkers of lipid transporters between the endoplasmic reticulum (ER)-to-Golgi traffic [29]. The categories of other downregulated genes include stress and oxyreduction, ATP binders, two cysteine proteinases (CP), cysteine synthase and a protein carrying myb transcription factor homology. Almost all of these genes are transcriptionally downregulated after 24 hours of compound treatment. The downregulation of heat shock proteins (Hsp 70 and Hsp 90), known to act as chaperones for signal transducers by blocking some steps of apoptotic pathways [30],[31], revealed the inhibition of any cell damage repair therefore impairing cell survival upon exposure to SF9B. Moreover, it is known that cysteine, a major thiol which replaces glutathione in *E. histolytica* and which plays a major role in growth and survival of *E. histolytica* [32],[33], is synthesized via a pathway consisting of two steps catalyzed by serine acetyltransferase and cysteine synthase [34],[35]. The reduced expression of cysteine synthase by treatment with SF9B implies low level of cysteine which is supposed to protect *E. histolytica* against oxidative stress and external environment that may cause cell death. Furthermore, down-regulation of two cysteine proteinases (CP-A5 and CP-A8) demonstrated loss or reduction of cytolytic activity of treated trophozoites. In fact, CP, especially CP-A5 was found to be associated with the trophozoite membrane and has been suggested to play a key role in host tissue invasion and destruction [36],[37].

**Table 5:** Changes in gene expression determined by RNASeq upon SF9B treatment of trophozoites.

Gene ID*	Description		
		Fold Change**	
		12 h	24 h
**Cellular organization/Cell growth**			
EHI_040600	Acid sphingomyelinase	nc	−2.16
EHI_131880	Acyl-coA synthetase	nc	−4.28
EHI_079300	Long-chain-fatty-acid–CoA ligase	−5.88	−9.90
EHI_140740	hypothetical protein (acyl-carrier)	nc	−3.31
EHI_073450	hypothetical protein (glycosyl transferase)	−6.71	−6.13
EHI_161070	hypothetical protein (lipid binding)	nc	−2.51
EHI_092490	hypothetical protein (synthase, fatty acid)	−8.83	−7.28
EHI_090430	hypothetical protein (synthase, fatty acid)	−18.81	−6.15
EHI_031640	hypothetical protein (synthase, fatty acid)	−6.68	−9.05
EHI_139080	Longevity-assurance family protein	nc	8.15
EHI_130860	Longevity-assurance family protein	5.5	4.53
**Stress and Oxyreduction**			
EHI_199590	70 kDa heat shock protein putative	nc	−4.38
EHI_163480	90 kDa heat shock protein	nc	−3.36
EHI_073480	ADP-ribosylation factor	nc	−3.66
EHI_189960	ADP-ribosylation factor	nc	−3.05
EHI_071590	Protein disulfide isomerase	nc	−1.97
EHI_021560	Thioredoxin	nc	−3.17
EHI_022950	DNAJ family protein	nc	−2.94
EHI_182520	DNAJ homolog subfamily A member 1	nc	−1.94
EHI_019630	hypothetical protein (Dipeptidyl-peptidase)	nc	−3.64
**Energy generation**			
EHI_095820	ATP-binding cassette protein	−5.39	−4.12
EHI_178050	ATP-binding cassette protein	−5.18	−3.91
EHI_131560	hypothetical protein (ATP-binding cassette)	−7.76	−4.18
EHI_170940	hypothetical protein	nc	−2.71
EHI_119510	hypothetical protein	nc	−3.45
EHI_117580	hypothetical protein	nc	−4.65
EHI_013340	hypothetical protein (Myb factor)	nc	−3.74
**Virulence**			
EHI_060340	Cysteine synthase A	nc	−2.03
EHI_010850	Cysteine proteinase A5	nc	−2.20
EHI_039610	Cysteine proteinase A8	nc	−2.26

Accession number from Amoeba DB data base, nc: non-significant changes.

Reads are the cDNA fragments sequenced and then mapped to the genome. Fold Change refers to the number of reads from cells in the tested condition divided by the number of reads from the non-treated control cells.

Overall the findings of the RNASeq experiment were reinforced by transcriptome analysis of *E. histolytica* treated in the presence of MTZ (92% of cell survival determined by trypan blue assay). The expression of the entire genes set of *E. histolytica* was examined using EH-IP2008 microarray [26]. By MTZ treatment, we highlighted the downregulation of peroxiredoxin encoding gene but no changes in ceramide biosynthesis genes were found (Table S3). Notice that genes whose transcription was modified by the presence of MTZ were not significantly modulated by SF9B treatment.

### SF9B treatment of *E. histolytica* modifies Gal/GalNAc lectin at the surface

**Figure 7:**
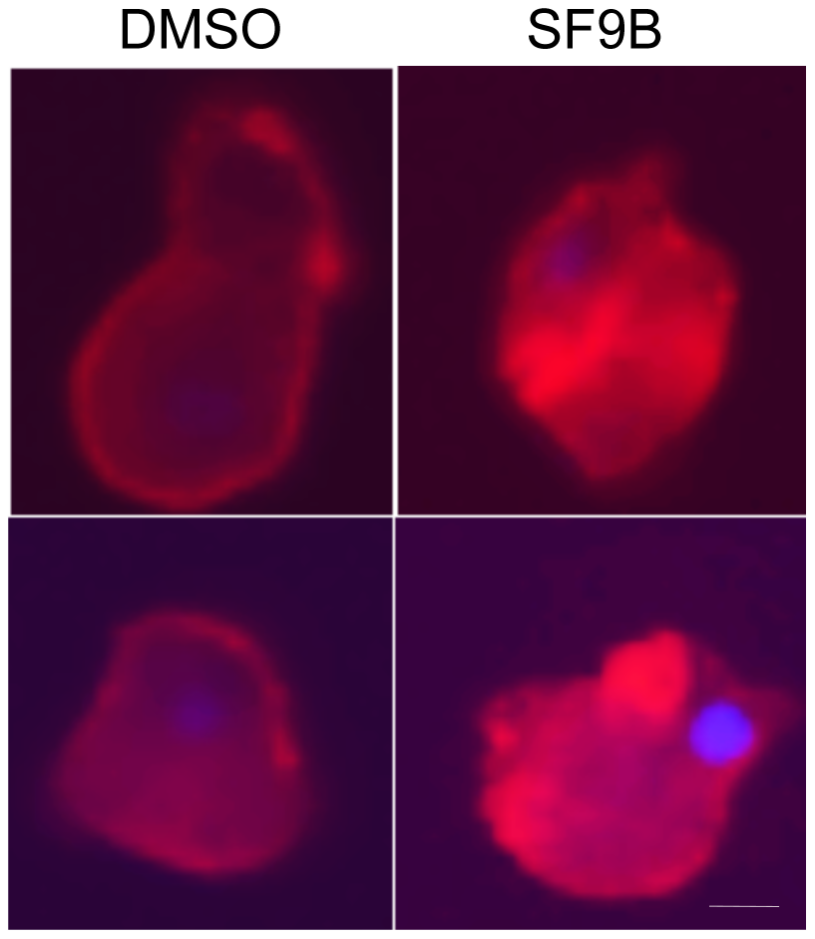
Localization of the Gal/GalNAc lectin on trophozoites surface after 12 hours treatment. DMSO is used as negative control and SF9B is tested at 2.75 µg/ml. The analysis was carried out with the epifluorescence microscope and photographs taken under 60× objective lens.

A dual role of acid shingomyelinase (ASM) has been suggested. First, it has an essential housekeeping function within the lysosomes and late endosomes of virtually all cells, participating in membrane turnover. Second, ASM translocates from intracellular compartments to the cell membrane, where hydrolysis of sphingomyelin into ceramide initiates membrane reorganization and facilitates the formation and coalescence of lipid microdomains, bringing inactive monomeric signalling proteins into active oligomers responsible for cell death. High levels of cholesterol and sphingolipid characterize these membrane domains. In *E. histolytica*, lipid rafts are enriched in Gal/GalNAc lectin, a surface protein complex involved in parasite adhesion to cells [38]. An immunofluorescence assay characterizing the Gal/GalNAc lectin, was thus used to examine the morphology changes and localization of the Gal/GalNAc lectin induced by active components from SF9B. In general, SF9B treatment caused significant changes on trophozoites surface after 12 hours of incubation. These changes were characterized by the accumulation of the cell surface Gal/GalNAc lectin in an agglomerate patches at certain points of the parasite surface (Figure 7).

## Discussion

In our previous study, fifty-five medicinal plants belonging to different families, selected on the basis of their traditional use against intestinal and liver disorders were tested for their anti-amoebic activities on polyxenic cultures of *E. histolytica* [19]. The aqueous extract from leaves of *C. variegatum* exhibited a clear anti-amoebic activity, therefore strengthening the local usage of this medicinal plant in the treatment of dysentery [19]. In the present research, the bioassay of extracts from different collection criteria aimed to search and adopt optimal conditions of phytochemical production in leaves of *C. variegatum*. The assays pointed out a variation of anti-amoebic activity between these aqueous extracts thereby demonstrating the potential environmental impacts on the production of phytochemicals in medicinal plants. In fact, plants contain and produce diverse secondary metabolites with interesting biological and pharmacological activities [3],[39] and the synthesis and accumulation of these secondary metabolites in medicinal plants are highly variable with many factors such as plant species, physiological development, geographical conditions, light exposure, soil conditions, season and harvest time [2],[40],[41]. It is known that stressed plants produce more secondary metabolites than non-stressed plants [42]. The collection sites in this study were in the same city, we did not find significant differences in anti-amoebic activity related to variable environmental conditions between extracts from the forest and the garden. The observation that anti-amoebic activity of leaves harvested in the morning and midnight was significantly higher than leaves harvested at other time of the day might be due to biological process taking place in the plant during the harvest. It is known that during the day, plants and especially leaves are undertaking photosynthesis (synthesis of primary metabolites) required for plant growth whereas in the night, they perform synthesis of secondary metabolites sometimes using the primary metabolites as precursors [43],[44]. While describing the fractionation process of compounds from the aqueous extract of *C. variegatum*, the anti-amoebic activity of each fraction or compound give a strong proof of the potency of this medicinal plant in the clearance of *in vitro* culture of *E. histolytica*.

The sub-fraction SF9B was identified for its strong anti-amoebic activity and when sub-fractionated, the resulting samples display reduced activity against *E. histolytica* suggesting that components of SF9B could act in synergy on single or multiple target sites associated with a physiological process or alternatively, the interaction between these compounds can improve their solubility and thereby enhance their bioavailability [45]. Synergic action has been noticed in several examples as a beneficial effect since it can help to eliminate eventual side effects associated with the predominance of a single xenobiotic compound in the body [46],[47]. In addition, it has been suggested that although phytotherapy do need isolation of active constituents, their identification is mostly required so that chemical profiling and establishment of phytoequivalence can be more precise in order to ensure herbal product approval and standardization [48].The modifications of the parasite morphology when treated with active SF9B suggest that the mechanism of action passes through destabilization of the cell membrane constituents. Moreover, the transcriptome analysis of treated parasites revealed reduction in level of ceramide through downregulation of acid shingomyelinase and several enzymes implicated in fatty acids production which supports the disorganization of the cell membrane. The up regulation of Lag genes, encoding proteins recently renamed ceramide synthase [49] might overcome the reduction of ceramide in treated amoebae therefore leading to high level of ceramide which may modify lipid rafts associated with Gal/GalNAc lectin and therefore activating signalling proteins into active oligomers responsible for programmed cell death and cell growth inhibition. This tentative hypothesis need to be further explored in *E. histolytica* but it can be supported by the fact that treatment of eukaryotic cells with several extracellular agents and stress stimuli (such as chemotherapeutic agents) have been found to be responsible for ceramide accumulation [50]. Ceramide accumulation most often occurs before any identifiable cell morphology changes, suggesting its important role in regulating programmed cell death [51]. On the other hand, programmed cell death has been reported in *E. histolytica* with morphological characteristics such as cell rounding, reduced cellular volume, nuclear condensation, DNA fragmentation and vacuolization of trophozoites [52]. Some of these markers have been noticed upon treatment with the sub-fraction SF9B, thereby suggesting that SF9B may modify the salvage pathway which utilizes long-chain sphingoid bases to form ceramide; alternatively a possible reduction of ceramide release may occur due to reduction fatty acid metabolism and lipids trafficking in *E. histolytica*.

### Conclusion

This study described a guided process of isolating compounds from the aqueous extract of *C. variegatum* which is active against trophozoites of *E. histolytica* growing on an axenic culture. A group of compounds was identified with strong anti-amoebic activity and their characterization and structure-activity modifications in the near future may identify a source of new anti-amoebic drugs. So far the most active sub-fraction SF9B induced substantial changes in cell morphology before leading to cell death. We hypothesize that the active components within SF9B might act through destabilization of cell architecture caused by changes in levels of ceramide, a membrane lipid involved in apoptosis and cell growth inhibition.

## Supporting Information

Table S1 Modulation of gene expression in *E. histolytica* upon 12 hours treatment with SF9B fraction. Genes that are identified as differentially expressed in treated amoebae (i.e. FDR adjusted p-value <0.05) in either DESeq or Cuffdiff analyses were listed. Fold-changes were shown in linear scale. “Average normalized read count” refers to the upper-quartile normalized read count averaged over the triplicates. “Average abundance in RPKM” refers to Reads Per Kilobase per Million mapped reads averaged over the triplicates.
(XLS)

Table S2 Modulation of gene expression in *E. histolytica* upon 24 hours treatment with SF9B fraction. Genes that are identified as differentially expressed in treated amoebae (i.e. FDR adjusted p-value <0.05) in either DESeq or Cuffdiff analyses were listed. Fold-changes were shown in linear scale. “Average normalized read count” refers to the upper-quartile normalized read count averaged over the triplicates. “Average abundance in RPKM” refers to Reads Per Kilobase per Million mapped reads averaged over the triplicates.
(XLS)

Table S3 Genes modulated in *E. histolytica* upon metronidazole treatment. Differences in gene expression were evaluated by microarray upon one hour treatment of trophozoites with 50 µM Metronidazole. Gene ID refers to the accession number of genes in AmoebaDB (http://amoebadb.org/amoeba/). FC: fold change; BY: False discovery rate by Benjamini and Yekutieli multiple testing; rawp: raw *p*-value.
(XLSX)
